# Supplementary material for: Investigating the influence of physiologically relevant hydrostatic pressure on CHO cell batch culture
Source: Sci Rep. 2021 Jan 8;11:162. doi: 10.1038/s41598-020-80576-8 (PMC7794228; doi:10.1038/s41598-020-80576-8)
Supplement: Supplementary file 1 — Supplementary Information 1. [file 41598_2020_80576_MOESM1_ESM.zip › S1/IgG/Jan17 standard curve/2/75ugml.pdf]

Data File C:\Users\P...s\ChemStation\1\Data\TK_CHO_IgG 2019-01-17 15-00-19\007-2-75ug_ml.D
Sample Name: 75ug/ml

=====================================================================

Acq. Operator : SYSTEM    Seq. Line : 7
Sample Operator : SYSTEM
Acq. Instrument : HPLC-0039    Location : 2
Injection Date : 1/17/2019 3:27:03 PM    Inj : 1
Inj Volume : 100.000 µl
Sequence File : C:\Users\Public\Documents\ChemStation\1\Data\TK_CHO_IgG 2019-01-17 15-00-19 \TK_CHO_IgG.S
Method : C:\Users\Public\Documents\ChemStation\1\Data\TK_CHO_IgG 2019-01-17 15-00-19 \CHO_IgG_LC.M (Sequence Method)
Last changed : 12/4/2018 1:38:41 PM by SYSTEM

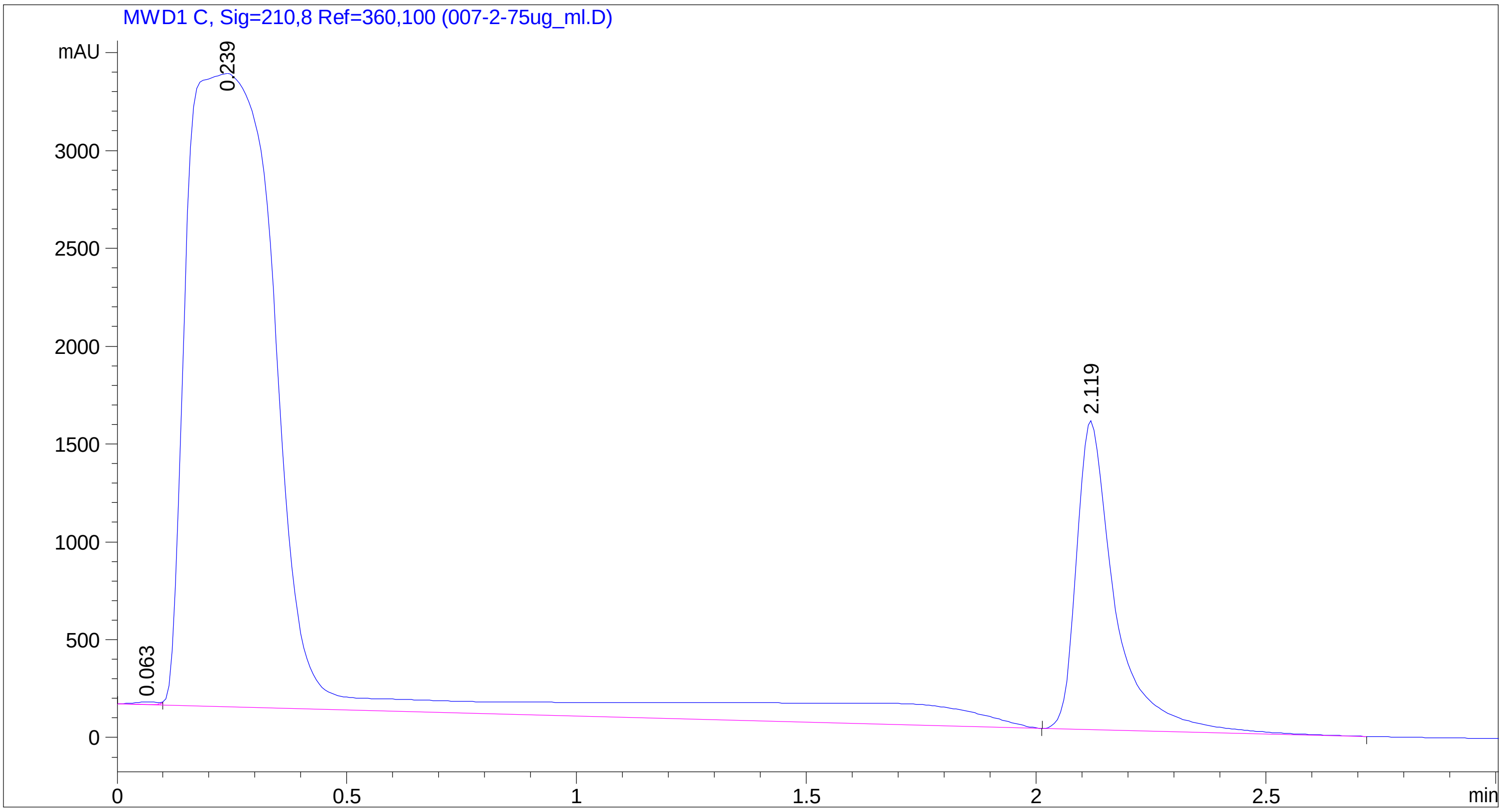


=====================================================================

Area Percent Report

=====================================================================

Sorted By : Signal
Multiplier : 1.0000
Dilution : 1.0000
Use Multiplier & Dilution Factor with ISTDs

Signal 1: MWD1 C, Sig=210,8 Ref=360,100

| Peak # | RetTime [min] | Type | Width [min] | Area [mAU*s] | Height [mAU] | Area % |
|---|---|---|---|---|---|---|
| 1 | 0.063 | BV E | 0.0497 | 41.21051 | 12.76536 | 0.0714 |
| 2 | 0.239 | VB R | 0.2167 | 4.84347e4 | 3234.82544 | 83.9618 |
| 3 | 2.119 | BBA | 0.0860 | 9210.68750 | 1580.98181 | 15.9668 |

Totals : 5.76866e4 4828.57261

=====================================================================

*** End of Report ***
